# Problematic Use of Internet-Related Activities and Perceived Weight Stigma in Schoolchildren: A Longitudinal Study Across Different Epidemic Periods of COVID-19 in China

**DOI:** 10.3389/fpsyt.2021.675839

**Published:** 2021-05-24

**Authors:** Xavier C. C. Fung, Andrew M. H. Siu, Marc N. Potenza, Kerry S. O'Brien, Janet D. Latner, Chao-Ying Chen, I-Hua Chen, Chung-Ying Lin

**Affiliations:** ^1^Department of Rehabilitation Sciences, Faculty of Health and Social Sciences, The Hong Kong Polytechnic University, Kowloon, Hong Kong; ^2^Departments of Psychiatry and Neuroscience and the Child Study Center, School of Medicine, Yale University, New Haven, CT, United States; ^3^Connecticut Council on Problem Gambling, Wethersfield, CT, United States; ^4^School of Social Sciences, Faculty of Arts, Monash University, Melbourne, VIC, Australia; ^5^Department of Psychology, University of Hawaii at Manoa, Honolulu, HI, United States; ^6^School of Physical Therapy and Graduate Institute of Rehabilitation Science, College of Medicine, Chang Gung University, Taoyuan, Taiwan; ^7^International College, Krirk University, Bangkok, Thailand; ^8^School of Education Science, Minnan Normal University, Zhangzhou, China; ^9^Institute of Allied Health Sciences, College of Medicine, National Cheng Kung University, Tainan, Taiwan; ^10^Department of Occupational Therapy, College of Medicine, National Cheng Kung University, Tainan, Taiwan; ^11^Department of Public Health, National Cheng Kung University Hospital, College of Medicine, National Cheng Kung University, Tainan, Taiwan

**Keywords:** COVID-19, weight stigma, problematic smartphone use, problematic social media use, children

## Abstract

**Background:** Social distancing and school suspension due to the coronavirus pandemic (COVID-19) may have a negative impact on children's behavior and well-being. Problematic smartphone use (PSU), problematic social media use (PSMU) and perceived weight stigma (PWS) are particularly important issues for children, yet we have a poor understanding of how these may have been affected by lockdowns and physical isolation resulting from COVID-19. This research aimed to understand how these psychosocial and behavioral variables may be associated with psychological distress, and how these associations may have changed during the COVID-19 pandemic.

**Methods:** A total of 489 children completed a three-wave longitudinal study from January 2020 to June 2020. The first wave was conducted before the COVID-19 outbreak. The second wave was conducted during the outbreak. The third wave was conducted during post-COVID-19 lockdown. Questionnaires measured psychological distress, PSU, PSMU, and PWS.

**Results:** PSU, PSMU, PWS and psychological distress were all significantly associated with each other. PSU was significantly higher during outbreak. PWS was significantly higher before outbreak. We found an increased association between PSMU and PWS across three waves in all three models. The association between PSU and depression/anxiety decreased across three waves; however, association between PSMU and depression/anxiety increased across three waves.

**Conclusions:** COVID-19 initiated school suspension and associated lockdowns appear to have exacerbated PSU and depression among children. However, PWS was reduced during this period. Children should use smartphones and social media safely and cautiously, and be aware of the potential exposure to weight stigmatization.

## Introduction

The COVID-19 pandemic is one of the greatest global public health concerns in living memory (1). The impact of the disease and approaches to its management go beyond the mortality and morbidity statistics, with the significant psychosocial impacts increasing recognized (2). There is growing evidence that the COVID-19 pandemic and associated containment measures (e.g., physical distancing and population lockdowns) may be causing psychological distress (3–5). Preventive measures such as physical/social distancing and forced lockdowns may underpin, at least in part, the reported increases in psychological distress (6). The social isolation during COVID-19 may also be driving changes in people's use, or overuse, of digital technologies, resulting in greater problematic smartphone use (PSU) and problematic social media use (PSMU) (7, 8).

Complying with the components model of addiction (9), PSU and PSMU are defined as the loss of control in smartphone and social media use, and their usage would lead to emotional changes, and difficulties in interpersonal relationships and study (10, 11). Other theories on PSU and PSMU [e.g., Interaction of Person-Affect-Cognition-Execution (I-PACE) model (12) and cognitive-behavioral model (13)] have proposed to indicate that inhibitory control, impulsive decision-making, high level of self-stigma, and poor self-esteem are key factors for PSU and PSMU development (14). PSU and PSMU among children have been of concern to clinicians and policy makers because of their purported association with children's psychological health, although the model of compensatory internet use suggests a *continuum* for smartphone and social media use (i.e., from non-problematic to problematic) (15). Indeed, several studies have suggested that PSU and PSMU are associated with greater depression, anxiety, and stress (16–21). The social distancing and school suspensions due to COVID-19 could possibly lead to undesirable lifestyle changes. Furthermore, it has been identified that boredom, loneliness, introversion, and preference for online interaction are risk factors for PSU and PSMU (10, 22). As children are forced into social distancing and accordingly spend more time indoors at home, they may be more likely to engage in PSU and PSMU as a means of distraction and/or way of engaging with others (8). As a result, children may experience greater psychological distress due to increased PSU and PSMU. However, there is little research on the potential impact of the COVID-19 pandemic on children's PSU and PSMU and psychological distress.

Aside from direct impacts on children's digital and social media use, children with overweight and obesity who regularly encounter perceived weight stigma (PWS) from others and accompanying psychological distress may be differentially affected by social distancing due to COVID-19. PWS is the perceived devaluation and discrimination directed toward people who are overweight or perceived to be overweight (23, 24). Any interpersonal relationship and media could be potential sources of PWS (25). However, prior evidence also shows that PWS exists in many individuals without overweight (24), and the negative effects of PWS on health for those without overweight are also serious (24, 26). Therefore, it is important to assess PWS among individuals across the weight spectrum (27). Social distancing may reduce exposure to in-person stigmatizing situations, but it could also result in different forms of stigma through the increased use of social media platforms (28, 29). Thus, it is important to investigate whether the outbreak of COVID-19 outbreak may have exacerbated existing psychological and behavioral concerns in vulnerable populations such as children who experienced weight-related stigma and/or internalized weight stigma. Children with overweight or obesity are at elevated risk of experiencing weight-related stigma, which may result in greater psychological impairments such as depression and anxiety (25, 30).

Given recent research suggesting that people with weight stigma are vulnerable to external stressors (31), it is possible that the COVID-19 outbreak may worsen psychological well-being in children who experienced higher weight stigma. In addition, it has been identified that experience of weight-based teasing, and exposure to media containing weight stereotype (e.g., cartoons or videos) are common sources that contribute to weight stigma (25). Therefore, it is reasonable to postulate that PSU and PSMU may be risk factors in weight stigma. Internet and social media could be the sources of weight-based stereotypes and stigmatization (32, 33). Increased PSU and PSMU may result in elevated exposure to weight-based stigmatization, from peers on social media or from information released to the public, potentially increasing children's PWS.

In this study, we proposed a conceptual model to investigate the relationship between PSU, PSMU, PWS and psychological distress, using three waves of longitudinal data spanning the COVID-19 pandemic. The relationships between these variables would be affected by changes in the stages of the COVID-19 pandemic. School children may have been affected by the suspension of teaching and associated lockdowns during COVID-19, and during the resumption of teaching post-lockdown. We used longitudinal data to examine changes in, and relationships between the variables of interest using three waves: (1) before COVID-19 outbreak, (2) during COVID-19 outbreak, and (3) post-COVID-19 lockdown and school suspensions. Specifically, we hypothesized that (1) PSU and PSMU would be positively associated with PWS and three forms of psychological distress (anxiety, depression, and stress); (2) PWS would be positively associated with three forms of psychological distress; (3) PSU, PSMU and PWS would be increased during the COVID-19 outbreak.

## Materials and Methods

### Participants and Procedure

This is a longitudinal study from January 2020 to June 2020. This study investigated psychological distress, weight stigma, and problematic internet-related behaviors among schoolchildren. Participants were recruited from three primary schools in Sichuan, China. Three waves of data collection were collected. Wave 1 was conducted before the COVID-19 outbreak, from January 1 to 9, 2020. Then the participants began to enter winter vacation and COVID-19 outbreak occurred during that time in mainland China. Wave 2 was conducted from March 1 to 9, 2020, during the outbreak of COVID-19. At that time, lockdown and online teaching were implemented, and thus children had experienced a lifestyle change in their society. The subsequent wave was conducted in June 2020 (i.e., Wave 3). As the COVID-19 was under control in that period, e.g., resumption of normal teaching in primary school, we define this is a post-lockdown period. Hence, this longitudinal study spanned three stages of the COVID-19 pandemic: (1) before outbreak, (2) during outbreak, and (3) post-lockdown. At Wave 1, 550 schoolchildren were approached and all agreed to participate in the first-wave study. At Wave 2, 543 of the 550 (response rate of 98.7%) agreed to participate in the second-wave study. At Wave 3, 489 of the 543 (response rate of 88.9%) agreed to participate in the third-wave study.

Participants were recruited with the assistance of the principals and teachers in three primary schools. Written informed consent was obtained from students and one of their parents. Paper survey was used for Wave 1. A set of questionnaires was distributed to students by teachers for completion in school. Due to the outbreak of COVID-19, we shifted the paper survey to online survey for Waves 2 and 3. The hyperlink to the questionnaires was sent to parents by the same teachers. As the format of survey changed, electronic informed consent was obtained on the first page of online survey. The questionnaires would appear after students selected “agree” on the first page.

There were three inclusion criteria: (1) children who were able to understand the questions, which were written in Mandarin; (2) they (or parents) had at least one smartphone with internet access and used it in the past month; and (3) they were in third to sixth grades. The only exclusion criterion for the present study was failure to participate in all three waves of surveys. At baseline, the participants' age range was between 9.80 and 13.68 years; weight range was between 24 and 85 kg; height range was between 105 and 165 cm; and body mass index (BMI) range was between 9.92 and 49.59 kg/m^2^. Moreover, we used BMI to define overweight with reference from 2005 mainland Chinese children at 85% percentile in BMI. Specifically, a boy was classified as having overweight when his BMI was higher than 17.0 (for 7–7.99 years), 17.7 (for 8–8.99 years), 18.4 (for 9–9.99 years), 19.4 (for 10–10.99 years), 20.1 (for 11–11.99 years), and 21.0 (for 12–12.99 years). A girl was classified as having overweight when her BMI was higher than 16.4 (for 7–7.99 years), 17.0 (for 8–8.99 years), 17.7 (for 9–9.99 years), 18.4 (for 10–10.99 years), 19.1 (for 11–11.99 years), and 20.0 (for 12–12.99 years) (34).

### Measures

#### Smartphone Application-Based Addiction Scale

The SABAS measures the level of PSU (9, 35). There are six items rated on a 6-point Likert scale from 1 (strongly disagree) to 6 (strongly agree). An example item for the SABAS is “During the past week, conflicts have arisen between me and my family (or friends) because of my smartphone use.” A higher score indicates a higher level of PSU. The Chinese version of SABAS has demonstrated good psychometrics properties (11, 36, 37). The internal consistency (Cronbach's α) of the SABAS in present study was 0.83 before outbreak, 0.88 during outbreak, and 0.89 in post-lockdown.

#### Bergen Social Media Addiction Scale

The BSMAS measures the level of PSMU (9, 35). There are six items rated on a five-point Likert scale from 1 (very rarely) to 5 (very often). An example item for the BSMAS is “How often during the last week have you felt an urge to use social media more and more?” A higher score indicates a higher level of PSMU. The Chinese version of BSMAS has demonstrated good psychometrics properties (11, 36, 37). The internal consistency (Cronbach's α) of the BSMAS in present study was 0.78 before outbreak, 0.90 during outbreak, and 0.88 in post-lockdown.

#### Perceived Weight Stigma Scale

The PWSS measures the level of PWS (38). Ten dichotomous questions (0 = no and 1 = yes) describe interpersonal discriminatory situations in different settings. An example item for the PWSS is “People act as if you are inferior because of your weight.” A higher score indicates a higher level of PWS. The Chinese PWSS has demonstrated good psychometric properties (29). The internal consistency (Cronbach's α) of the PWSS in present study was 0.87 before outbreak, 0.84 during outbreak, and 0.80 in post-lockdown.

#### Depression, Anxiety, Stress Scale-21

The DASS-21 measures the level of psychological distress (39). There are 21 items rated on a four-point Likert scale (0 = never; 1 = sometimes; 2 = often; 3 = almost always). It consists of three subscales (seven items each): stress, anxiety, and depression. Example items are “I felt that I was using a lot of nervous energy” for stress; “I felt I was close to panic” for anxiety; “I found it difficult to work up the initiative to do things” for depression. A higher score indicates a higher level of psychological distress. In this study, cut-off scores for mild depression (score higher than 10), anxiety, (score higher than 8) and stress (score higher than 15) were used to define whether a student has depression, anxiety, and stress (40). The Chinese DASS-21 has demonstrated good psychometric properties (41). The internal consistency (Cronbach's α) of the DASS-21 in present study was 0.93 before outbreak, 0.91 during outbreak, and 0.93 in post-lockdown.

### Data Analysis

The repeated measures analysis of variance (ANOVA) was used to examine the differences in PSU, PSMU, and PWS among the three waves (i.e., before outbreak, during outbreak, and post-lockdown). Chi-square test was used to examine the differences in the numbers of mild depression, stress, and anxiety (detailed information of the cutoffs for mild depression, stress, and anxiety described in Depression, Anxiety, Stress Scale-21) among the three waves. Pearson's correlation was used to examine the relationships between PSU, PSMU, PWS, depression, anxiety, and stress in each wave.

SEM with multiple group analysis was used to examine the fit of the three proposed models across three waves. The χ^2^ test and the following fit indices were used to determine the fitness of the models: comparative fit index (CFI), root mean square error of approximation (RMSEA), non-normed Fit Index (NNFI), and the standardized root mean square residual (SRMR). The models would be considered as acceptable if there is a non-significant χ^2^, CFI and NNFI > 0.9, RMSEA and SRMR < 0.08.

All the analyses were performed using IBM SPSS 24.0 (IBM Crop., Armonk, NY), except for SEMs, which was performed using LISREL 8.80 (Scientific Software International, Chicago, IL).

## Results

All the 489 participants provided valid data for statistical analyses and their demographic information is shown in Table 1. Participants' mean age was 11.6 years. Notably, the body mass index was significantly different across the three waves (18.84 before outbreak; 20.16 during outbreak; 19.54 in post-lockdown).

**Table 1:** Characteristics of participants (*N* = 489).

	***M*** **(*****SD*****) or** ***n*** **(%)**			***F* or χ^**2**^**
	**Before outbreak**	**COVID-19 outbreak**	**Post-lockdown**	
Age (year)	11.60 (0.74)	11.60 (0.73)	11.61 (0.74)	0.01
Gender (Boy)	247 (51%)	247 (51%)	247 (51%)	
Height (cm)	145.54 (8.60)	145.74 (8.60)	147.45 (8.82)	17.94^**^
Weight (kg)	39.61(11.45)	42.87(14.10)	42.44(12.10)	26.77^**^
Body Mass Index. (kg/m^2^)	18.84(5.79)	20.16(6.45)	19.54(5.68)	20.90^**^
Objective weight status (Overweight)	124(25.35)	161(32.92%)	157(32.11%)	5.59
Perceived weight status (Overweight)	78(15.95%)	83(16.97%)	85(17.38%)	0.32

* *p < 0.05*;

** *p < 0.01*;

*** *p < 0.001*.

*Objective weight status indicates overweight defined using BMI with reference from 2005 mainland Chinese children at 85% percentile in BMI (So et al., 2008). Perceived weight status indicates overweight defined using the question item “What do you think your weight status is?” with responses of “Very thin,” “Thin,” “Normal weight,” “Overweight,” and “Obese.”*

The score on PSU, PSMU, PWS, and the number of participants who experienced depression, anxiety, and stress are shown in Table 2. PSU during the COVID-19 outbreak was significantly higher than the score before the outbreak, but not significantly different from post-lockdown. PSMU scores were significantly different across the three waves; however, *post-hoc* analysis indicated no significant difference between any pair. PWS before the outbreak was significantly higher than in the two subsequent waves. As for psychological distress, participants were more likely to experience depression during the outbreak compared to post-lockdown; more likely to experience anxiety before the outbreak compared to during the outbreak; and more likely to experience stress during the outbreak and post-lockdown compared to before the outbreak.

**Table 2:** Comparisons of problematic internet-related use, perceived weight stigma, fear of COVID-19, and psychological distress across time.

	***M*** **(*****SD*****) or** ***n*** **(%)**			***F* or χ^**2**^ (*p*-value)**	***Post hoc***
	**Before outbreak**	**COVID-19 outbreak**	**Post-lockdown**		
	***n* = 489**	***n* = 489**	***n* = 489**		
PSU	1.64 (0.85)	1.78 (0.90)	1.71 (0.89)	4.79 (0.01)	2>1,2=3
PSMU	1.41 (0.55)	1.38 (0.60)	1.34 (0.55)	2.77 (0.06)	–
PWS	0.22 (0.24)	0.11 (0.19)	0.11 (0.22)	63.13 (<0.01)	1>2,3
Depression (Yes)^a^	81 (16.6)	90 (18.4)	70 (14.3)	1 vs. 2: 0.73 (0.39) 1 vs. 3: 1.07 (0.31) 2 vs. 3: 3.92 (0.04)	–
Anxiety (Yes)^a^	161 (32.9)	137 (28.0)	141 (28.8)	1 vs. 2: 3.64 (0.05) 1 vs. 3: 2.35 (0.12) 2 vs. 3: 0.10 (0.74)	–
Stress (Yes)^a^	62 (12.7)	89 (18.2)	91 (18.6)	1 vs. 2: 6.81 (0.01) 1 vs. 3: 6.95 (0.01) 2 vs. 3: 0.04 (0.83)	–

*PSU, Problematic smartphone use; PSMU, Problematic social media use; PWS, Perceived weight stigma*.

a *Depression, anxiety, and stress were defined using cutoff scores in the Depression, Anxiety, and Stress Scale (DASS-21) on mild depression, anxiety, and stress, respectively*.

Table 3 shows the correlation between variables. All the correlations were significant across the three epidemic situations. In all three waves, variables were positively correlated with each other, ranging from 0.32 to 0.82 before the outbreak, from 0.31 to 0.78 during the outbreak, and from 0.19 to 0.81 in post-lockdown.

**Table 3:** Correlation matrix between studies variables (*N* = 489).

	***r*** **(*****p*****-value)**					
	**1**.	**2**.	**3**.	**4**.	**5**.	**6**.
**Before outbreak**						
1. PSU	–					
2. PSMU	0.505 ^**^	–				
3. PWS	0.321^**^	0.234^**^	–			
4. Depression	0.504^**^	0.379^**^	0.427^**^	–		
5. Anxiety	0.407^**^	0.311^**^	0.409^**^	0.751^**^	–	
6. Stress	0.422^**^	0.372^**^	0.462^**^	0.820^**^	0.815^**^	–
**COVID-19 outbreak**						
1. PSU	–					
2. PSMU	0.633^**^	–				
3. PWS	0.313^**^	0.356^**^	–			
4. Depression	0.449^**^	0.501^**^	0.475^**^	–		
5. Anxiety	0.428^**^	0.483^**^	0.483^**^	0.732^**^	–	
6. Stress	0.504^**^	0.520^**^	0.501^**^	0.777^**^	0.768^**^	–
**Post-lockdown**						
1. PSU	–					
2. PSMU	0.651^**^	–				
3. PWS	0.188^**^	0.216^**^	–			
4. Depression	0.357^**^	0.382^**^	0.387^**^	–		
5. Anxiety	0.372^**^	0.402^**^	0.418^**^	0.764^**^	–	
6. Stress	0.491^**^	0.436^**^	0.380^**^	0.747^**^	0.811^**^	–

*PSU, Problematic smartphone use; PSMU, Problematic social media use; PWS, Perceived weight stigma*.

* *p < 0.05*;

** *p < 0.01*;

*** *p < 0.001*.

**Figure 1:**
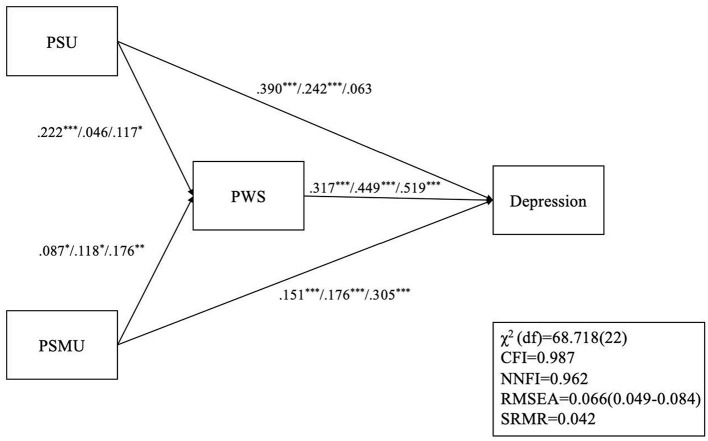
Structural equation modeling with multiple group analysis on the relationship among PSU, PSMU, PSW, and depression. *p < 0.05; **p < 0.01; ***p < 0.001; For each set of path coefficients, the first coefficient represents pre-COVID associations (Wave 1); the second coefficient represents associations during the COVID outbreak (Wave 2); the last coefficient represents post-COVID lockdown (Wave 3) associations; PSU, problematic smartphone use; PSMU, Problematic social media use; PWS, perceived weight stigma; CFI, Comparative fit index; NNFI, non-normed fit index; RMSEA, root mean square error of approximation; SRMR, standardized root mean square residual.

The depression model (Figure 1) had satisfactory model fit except for the χ^2^ (χ^2^[*df* ] = 68.718 [22]; *p* < 0.001), CFI = 0.987, NNFI = 0.962, RMSEA = 0.066, and SRMR = 0.042. For before the outbreak, all paths were significant. For the outbreak period, only the path between PSU and PWS was not significant. In post-lockdown, only the path between PSU and depression was not significant.

**Figure 2:**
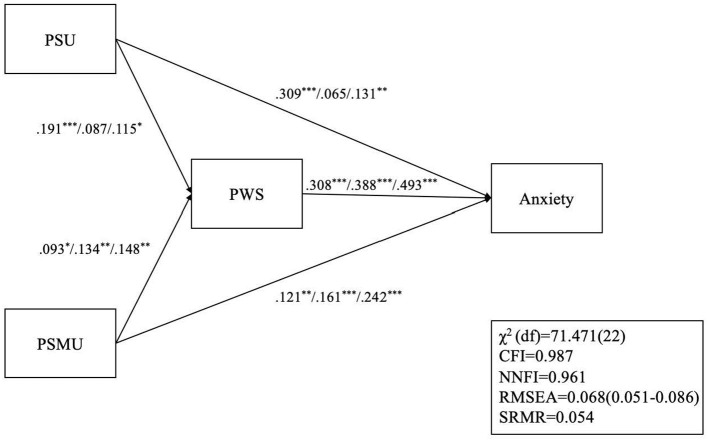
Structural equation modeling with multiple group analysis on the relationship among PSU, PSMU, PSW, and anxiety. *p < 0.05; **p < 0.01; ***p < 0.001; For each set of path coefficients, the first coefficient represents pre-COVID associations (Wave 1); the second coefficient represents associations during the COVID outbreak (Wave 2); the last coefficient represents post-COVID lockdown (Wave 3) associations; PSU, problematic smartphone use; PSMU, Problematic social media use; PWS, perceived weight stigma; CFI, Comparative fit index; NNFI, non-normed fit index; RMSEA, root mean square error of approximation; SRMR, standardized root mean square residual.

The anxiety model (Figure 2) had satisfactory model fit except for the χ^2^ (χ^2^[*df* ] = 71.471 [22]; *p* < 0.001), CFI = 0.987, NNFI = 0.961, RMSEA = 0.068, and SRMR = 0.054. All paths were significant for before the outbreak as well as in post-lockdown. However, two paths were not significant during the outbreak, which are the path between PSU and PWS, and the path between PSU and anxiety.

**Figure 3:**
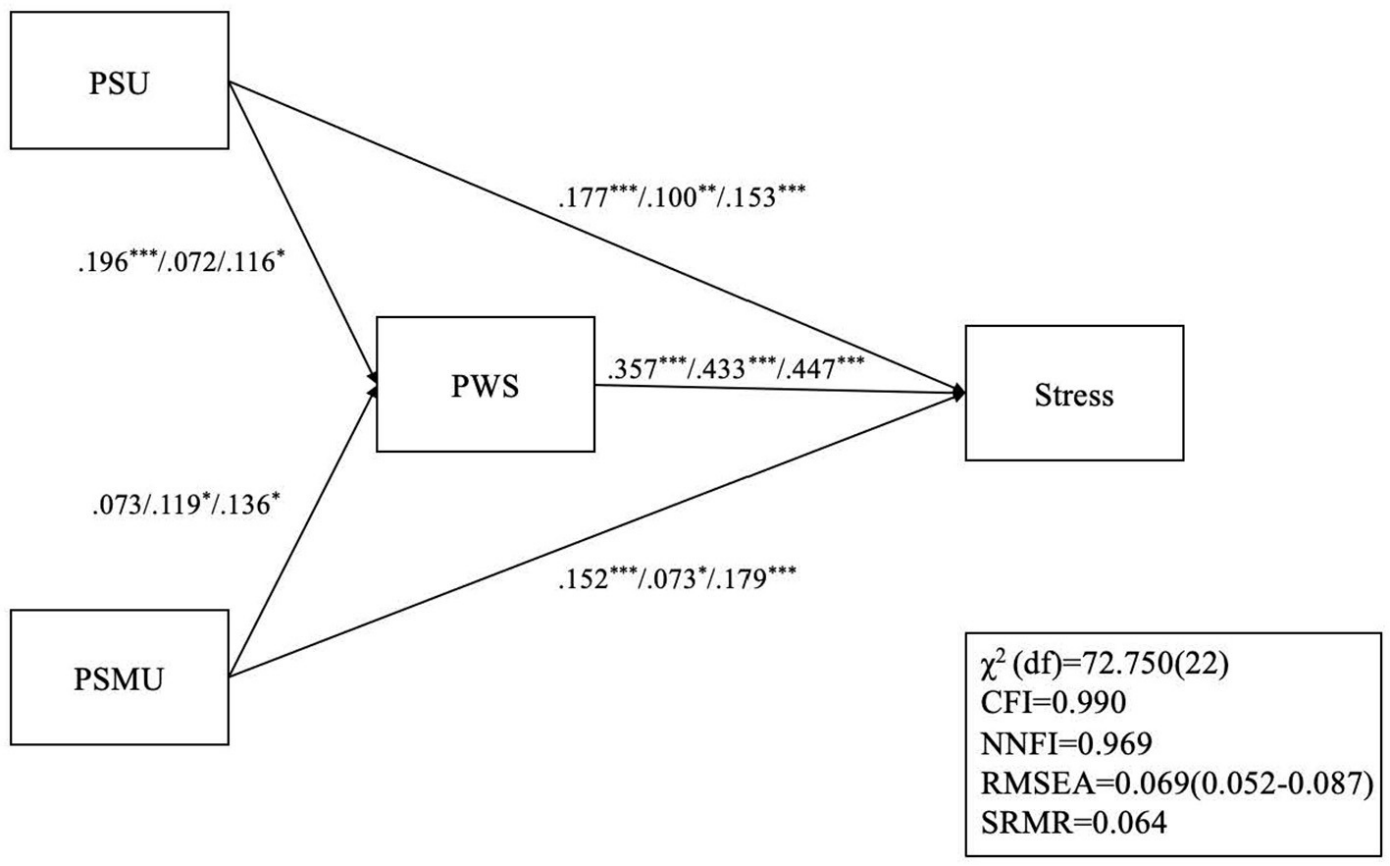
Structural equation modeling with multiple group analysis on the relationship among PSU, PSMU, PSW, and stress. *p < 0.05; **p < 0.01; ***p < 0.001; For each set of path coefficients, the first coefficient represents pre-COVID associations (Wave 1); the second coefficient represents associations during the COVID outbreak (Wave 2); the last coefficient represents post-COVID lockdown (Wave 3) associations; PSU, problematic smartphone use; PSMU, Problematic social media use; PWS, perceived weight stigma; CFI, Comparative fit index; NNFI, non-normed fit index; RMSEA, root mean square error of approximation; SRMR, standardized root mean square residual.

The stress model (Figure 3) also had satisfactory model fit except for the χ^2^ (χ^2^[*df* ] = 72.750 [22]; *p* < 0.001), CFI = 0.990, NNFI = 0.969, RMSEA = 0.069, and SRMR = 0.064. For the pre-outbreak period, only the path between PSMU and PWS was not significant. For the outbreak period, only the path between PSU and PWS was not significant. All paths were significant in post-lockdown.

## Discussion

We investigated the relationships between PSU, PSMU, PWS, and three forms of psychological distress (depression, anxiety, stress) across three waves with the use of SEM. All the models had satisfactory model fit. PSU and PSMU had significant effects on PWS and psychological distress. Additionally, PWS had a significant effect on psychological distress. Furthermore, we found that PSU was significantly higher during the COVID-19 outbreak, while the PWS was significantly higher before the COVID-19 outbreak. However, there was no significant difference in PSMU across the three waves.

The lower level of PWS during COVID-19 outbreak and post-lockdown may be explained by the diminished social interaction, and hence, fewer chances to be stigmatized. Mainly, children experienced weight-based teasing and bullying at school (42–44). Research showed that children who experienced weight-based teasing were likely to perform avoidance behaviors such as skipping school, suggesting that escaping social interaction could be a relief (44). It does not appear that other sources of stigma such as through social media made up for this reduced experience of stigma. As the face-to-face teaching was suspended during the COVID-19 outbreak, interactions among children were reduced to a minimum. The decreased social interaction may also explain the lower rates of anxiety during the COVID-19 outbreak, possibly due to less anxiety-provoking social interactions with peers, including lower weight stigma, and less academic-related anxiety while school was not meeting in person. However, depression and stress were still significantly higher during the outbreak than before the outbreak.

We found an increased association between PSMU and PWS across the three waves in all three models. Results are consistent with studies on social media and weight stigma, which indicated social media could be a source of stigmatization (32, 33). Thus, it is possible that children may have suffered from weight stigma without face-to-face interaction. Parents, teachers, and healthcare providers should be aware of the stigmatization from social media.

The association between perceived weight stigma and psychological distress concurs with the findings of other research on weight stigma (25, 29, 30, 45). A recent meta-analysis (30) found that perceived weight stigma was significantly associated with anxiety and depression in eight empirical studies. Similarly, perceived weight stigma was negatively associated with mental health in another recent meta-analysis (45). Furthermore, our results were consistent with research on young adults from the United States, which indicated that prior experience of weight stigma was a predictor of higher levels of stress (β = 0.15) and depressive symptoms (β = 0.15) during the COVID-19 pandemic (46). However, we found stronger associations between PWS and stress (β = 0.357–0.447), and PWS and depression (β = 0.317–0.519). Apart from the difference in measures, a possible explanation for the stronger associations is that children were possibly more vulnerable to weight stigma and its repercussions compared to young adults (47).

Moreover, PWS was reduced during the COVID-19 outbreak; however, the association between PWS and psychological distress seems to have been exacerbated. It may imply the lingering effects of stigma. For instance, research has indicated that self-esteem did not rebound after body mass reduced to normal range (48) and that weight stigma can linger even after weight loss is achieved (49). Although the PWS may have been reduced, the negative psychological consequences caused by PWS appear to be difficult to overcome.

On the other hand, our findings on the increased PSU during COVID-19 concurs with a German study, which indicated increased screen time among children during COVID-19 (50). Furthermore, we found positive associations between PSU/PSMU and psychological distress. However, their associations changed direction across the three waves. Specifically, the association between PSU and depression/anxiety decreased from before, during, to post-lockdown; however, association between PSMU and depression/anxiety increased from before, during, to post-lockdown. The diminished associations between PSU and depression/anxiety may be due to the recently designed mental health apps (51–53). Although we did not ask whether our participants used such apps, it could be possible that participants used apps to cope with their depression and anxiety. The exacerbated associations between PSMU and depression/anxiety may be due to the rumors and frightening news or information on COVID-19 in the social media (54, 55).

Results of the current study underscore the problematic nature of weight-based stigmatization and its psychological consequences in youth. Parents and teachers should remain attentive to children's interaction with peers. Communication between parents and teachers is vital to understanding children's interpersonal relationships and staying attuned to possible instances of stigmatization or bullying. Such vigilance will allow for timely education and intervention. Moreover, while school suspension and online teaching may be inevitable in the pandemic, it is necessary to monitor children smartphone and social media use. For instance, parents may formulate a timetable for children to balance study time and leisure time.

There were some limitations to this study. First, fear of the pandemic itself could be a source of psychological distress. Including anxiety about COVID-19 as a variable in future studies might provide better insight (3). Second, self-reported measures were used. The responses might not be accurate due to recall bias and social desirability bias. Lastly, we only recruited participants from Sichuan Province of mainland China; therefore, the representativeness of the present study's sample might not be generalizable to the entire mainland China.

## Conclusion

The current longitudinal study investigated the relationships between PSU, PSMU, PWS, and psychological distress across three stages of the COVID-19 pandemic: before outbreak, during outbreak, and post-lockdown. Results suggested that PSU, PSMU, PWS were associated with depression, anxiety, and stress; PSU and PSMU were associated with PWS. Furthermore, PWS was reduced during school closures and lockdowns, however, its negative impact on psychological distress seems persistent. Moreover, social media is another important source of stigmatization that deserves further investigation. As PSU and PSMU were associated with PWS and psychological distress, parents and teachers are encouraged to educate children about using smartphones and social media safely and cautiously. In particular, families must be made aware of their psychological health consequences and the potential exposure to weight stigmatization.

## Data Availability Statement

The raw data supporting the conclusions of this article will be made available by the authors, without undue reservation.

## Ethics Statement

The studies involving human participants were reviewed and approved by the research proposal was approved by the Ethics Committee of the Hong Kong Polytechnic University's ethics committee (IRB ref: HSEARS20190718001) and the Institutional Review Board of the Jianxi Psychological Consultant Association (IRB ref: JXSXL-2020-J013). Written informed consent for participation was not provided by the participants' legal guardians/next of kin because: The study period involves lockdown period and the physical contact was not allowable. Therefore, we obtained online consent instead of written consent.

## Author Contributions

XF, AS, MP, KO'B, JL, I-HC, and C-YL contributed conception and design of the study. XF and I-HC organized the database. I-HC and C-YL performed the statistical analysis. AS, MP, KO'B, JL, and C-YC interpreted the results. XF wrote the first draft of the manuscript. C-YC, I-HC, and C-YL wrote sections of the manuscript. All authors contributed to manuscript revision, read and approved the submitted version.

## Conflict of Interest

The authors declare that the research was conducted in the absence of any commercial or financial relationships that could be construed as a potential conflict of interest.
